# Class III treatment using facial mask: Stability after 10 years

**DOI:** 10.1590/2176-9451.19.5.123-135.bbo

**Published:** 2014

**Authors:** Adilson Luiz Ramos

**Affiliations:** 1 PhD in Orthodontics, State University of São Paulo (UNESP) / Araraquara. Adjunct professor, Department of Dentistry, State University of Maringá (UEM). Professor, Postgraduate program, Brazilian Dental Association (ABO), UNICESUMAR and UEM

**Keywords:** Angle Class III malocclusion, Face mask, Stability

## Abstract

Early Class III malocclusion treatment may not have long-term stability due to mandibular growth. Although some features of this malocclusion point to a better prognosis, it is practically impossible for the orthodontist to foresee cases that require new intervention. Many patients need retreatment, whether compensatory or orthodontic-surgical. The present study reports the case of a Class III patient treated at the end of the mixed dentition with the use of a face mask followed by conventional fixed appliances. The case remains stable 10 years after treatment completion. It was presented to the Brazilian Board of Orthodontics and Dentofacial Orthopedics (BBO) as a requirement for the title of certified by the BBO.

## Abstract (pt)

O tratamento precoce da má oclusão de Classe III pode não apresentar estabilidade em longo prazo, em decorrência do crescimento mandibular. Embora algumas características sinalizem um melhor prognóstico para o tratamento dessa má oclusão, é praticamente impossível para o ortodontista prever qual caso irá requerer nova intervenção. Muitos pacientes precisam de retratamento, que pode ser compensatório ou mesmo ortodôntico-cirúrgico combinado. O presente artigo relata o tratamento realizado em um paciente com má oclusão de Classe III, no final da dentição mista, mediante o auxílio da máscara facial seguida de aparelhagem fixa convencional, e que continua estável após 10 anos da conclusão do tratamento. Esse caso foi apresentado à Diretoria do Board Brasileiro de Ortodontia e Ortopedia Facial (BBO) como parte dos requisitos para obtenção do título de Diplomado pelo BBO.

## INTRODUCTION

This study reports the case of a 12-year and 4-month-old patient referred to treatment with chief complaint of "crossed front teeth and protruded lower lip".The patient sought improvements in smile and facial esthetics. He was in good general health without relevant register in his medical history, and presented in regular oral hygiene with a few white spot lesions and mild gingivitis. His parents reported that anterior crossbite was not present in deciduous dentition, it only appeared after permanent incisors eruption.

## DIAGNOSIS

**Figure 1:**
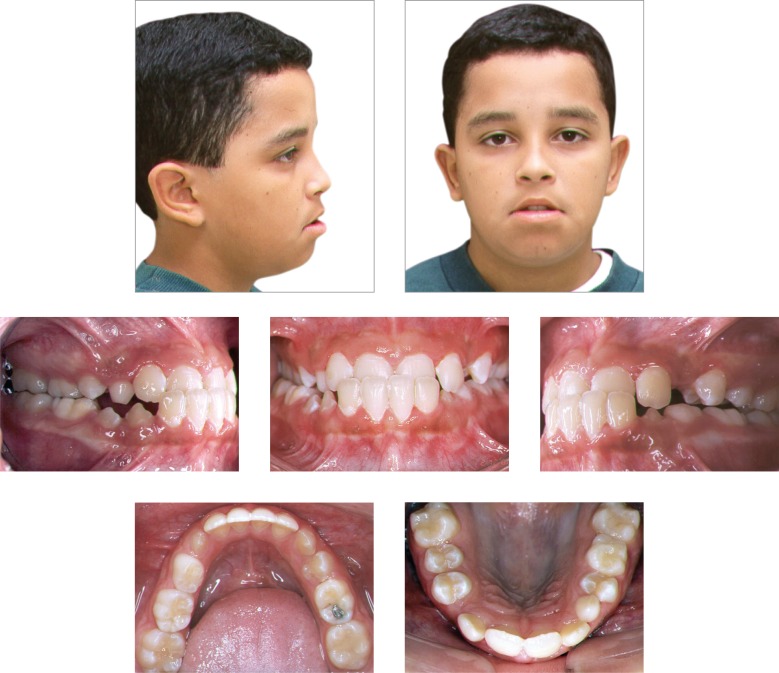
Initial facial and intraoral photographs

**Figure 2:**
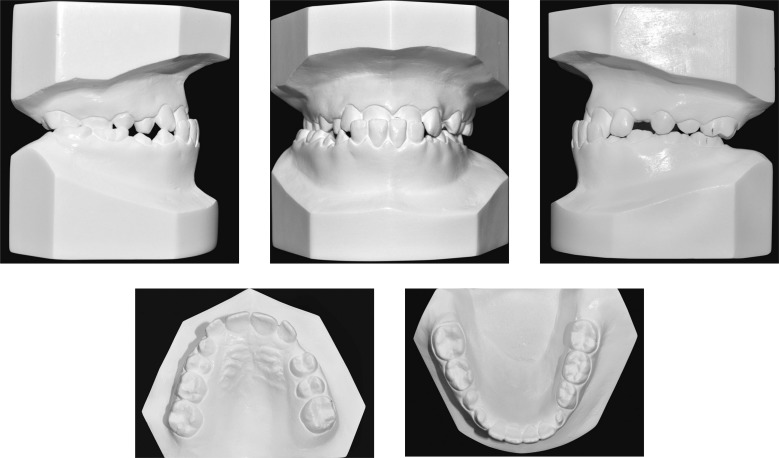
Initial casts.

Facial assessment (Fig 1) revealed absence of passive labial seal, a concave profile and protruded lower lip; thereby suggesting anteroposterior skeletal relationship (Class III).The patient was at the end of the second transitional period of the mixed dentition with Class III molar relationship (Figs 1 and 2).He presented unbalanced maxillomandibular transverse relationship which resulted in functional unilateral posterior crossbite from tooth #12 to #16 and lower midline deviation to the right when in maximum intercuspation.

**Figure 3:**
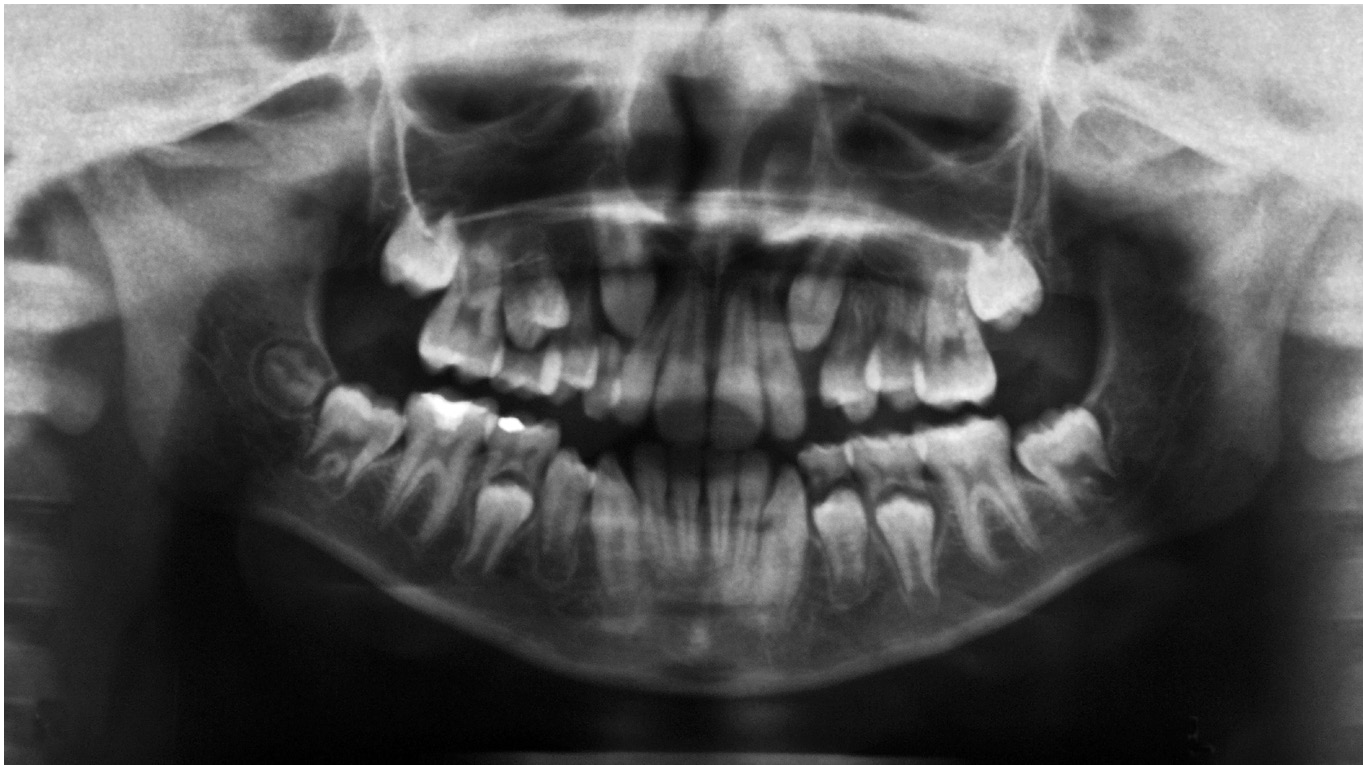
Initial panoramic radiograph.

**Figure 4:**
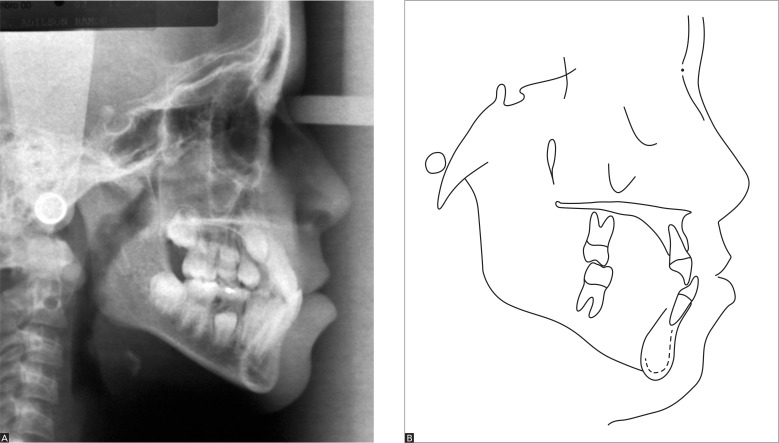
Initial lateral cephalogram (A) and cephalometric tracing (B).

Panoramic radiograph (Fig 3), taken at the end of the second transitional period of the mixed dentition, did not reveal any abnormalities. Cephalometric analysis (Fig 4 and Tab 1) highlighted retrusion of the maxilla (SNA = 80^o)^ and poor maxillomandibular relationship for the patient's age (ANB = 0.5^o)^.He had retruded retroclined maxillary incisors (1-NA = 18^o ^and 2 mm) as well as protruded and buccally tipped mandibular incisors (1-NB = 29^o^ and 7 mm). These dental features indicated dentoalveolar and skeletal components of Class III malocclusion.

**Table 1:** Initial (A), final (B) and control cephalometric values 10 years after treatment (C).

	Measurements		Normal	A	B	Dif. A/B	C
**Skeletal pattern**	SNA	(Steiner)	82°	80°	82°	2°	82°
	SNB	(Steiner)	80°	79.5°	80°	0.5°	81°
	ANB	(Steiner)	2°	0.5°	2°	1.5°	1°
	Angle of convexity	(Downs)	0°	2°	3°	1°	2.5°
	Y-Axis	(Downs)	59°	63°	61.5°	1.5°	62°
	Facial angle	(Downs)	87°	87°	89.5°	2.5°	89
	SN-GoGn	(Steiner)	32°	35°	32°	3°	32°
	FMA	(Tweed)	25°	33°	28°	5°	28°
**Dental ** **pattern**	IMPA	(Tweed)	90°	92.5°	97.5°	5°	97°
	1.NA (degrees)	(Steiner)	22°	18°	29°	11°	28°
	1-NA (mm)	(Steiner)	4 mm	2 mm	5 mm	3 mm	5°
	1.NB (degrees)	(Steiner)	25°	29°	32°	3°	30°
	1-NB (mm)	(Steiner)	4 mm	7 mm	6 mm	1 mm	6 mm
	- Interincisal angle	(Downs)	130°	131°	115.5°	15.5°	120°
	1-APo	(Ricketts)	1 mm	6 mm	5 mm	1 mm	5 mm
**Profile**	Upper lip — S-line	(Steiner)	0 mm	-0.5 mm	0 mm	0.5 mm	0 mm
	Lower lip — S-line	(Steiner)	0 mm	-5 mm	-4 mm	1 mm	-4 mm

In functional terms, there was little anterior mandibular shift to the right from centric relation to maximum intercuspation.

## TREATMENT PLAN

Treatment planning initially aimed at correcting maxillomandibular discrepancy by means of a face mask followed by rapid expansion of the maxilla which also contributed to correct the transverse deficiency. To this end, a modified Haas appliance associated with hooks in the region of canines used as support for protraction of the maxilla was used. Patient and parents were aware of the need for strong compliance to achieve treatment success. They were also informed that unpredictable mandibular growth could create the need for a new intervention and potential orthognathic surgery during adulthood.

Thus, they were presented with an alternative approach: wait for growth completion during adolescence and have orthodontic treatment with fixed appliances and potential orthognathic surgery carried out in the future. Patient's parents agreed on the early approach and follow-up to monitor the possibility of new interventions. A pre-adjusted, metallic, fixed orthodontic Roth prescription appliance would be installed in both maxillary and mandibular arches. After treatment finishing, the retention phase would begin with the use of a removable wraparound retainer in the maxillary arch and an intercanine bar in the mandibular arch.

## TREATMENT PROGRESS

**Figure 5:**
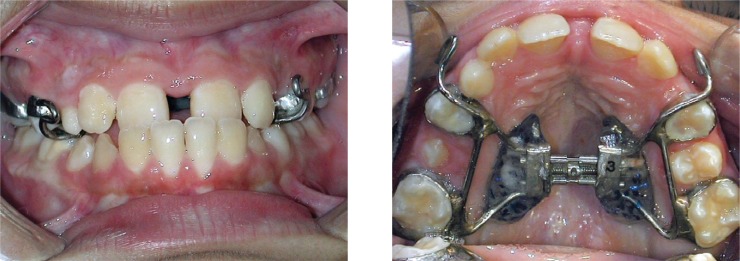
Treatment outcomes after 14 days using the expander activated twice a day.

**Figure 6:**
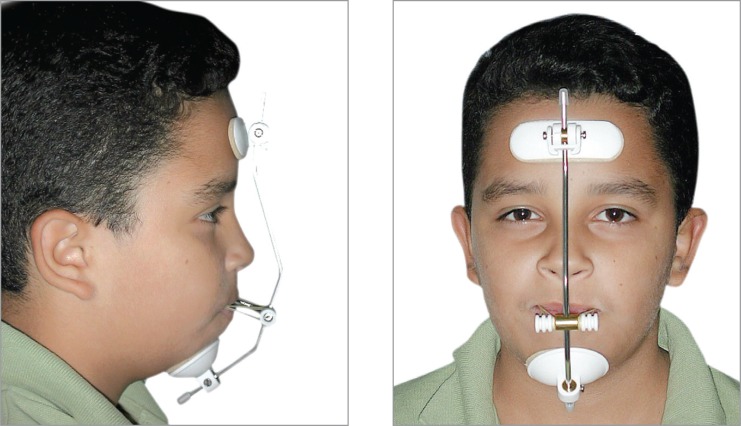
Petit face mask with 600-g force applied.

The modified Haas appliance was manufactured based on a Hyrax expander. Maxillary expansion followed an activation protocol of 1/4 turn every 12 hours for 14 days. Figure 5 illustrates the final outcomes. After 14 days, the expansion appliance remained stabilized and therapy with Petit face mask began (Fig 6). A 600-g force was applied 14 hours a day for 6 to 8 months so as to overcorrect overjet. However, the patient reported having applied force 12 hours a day and, for this reason, 6 months were rendered necessary to correct anterior crossbite. He was then advised to wear the appliance while sleeping for 4 months so as to achieve protraction stability.

**Figure 7:**
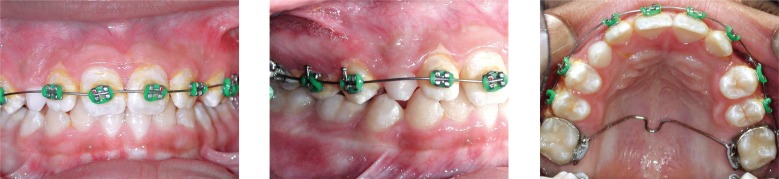
Alignment and leveling onset in the upper arch with palatal bar used for transverse maintenance and control of first molars rotation. Fluoride-varnish was applied every three months to minimize the incidence of white spot lesions.1

After removing the expander and discontinuing protraction, a removable 0.8-mm stainless steel wire palatal bar was installed to achieve transverse stability and adjust first molars rotation. During the same appointment, pre-adjusted, metallic, fixed orthodontic Roth prescription brackets were bonded on maxillary teeth, except for unerupted canines. Alignment and leveling procedures began with the use of 0.016-in NiTi archwire followed by 0.016, 0.018 and 0.020-in stainless steel archwire associated with omega loops on molars mesial surface until canines erupted so as to preserve dental arch circumference. At treatment finishing, 0.019 x 0.025-in stainless steel rectangular archwire was used. Fluoride-varnish at 5 ppm (Duraphat^(r)^, Colgate, Germany) was applied every three months to prevent white spot lesions^1^ (Fig 7).

On the mandibular arch, bracket bonding was carried out with individualized angulation for teeth #43 and 33 so as bracket slots were perpendicular to the root axis, thereby favoring format compensation. The sequence of archwires used for alignment and leveling was similar to that used in the maxillary arch: 0.019 x 0.025-in stainless steel rectangular archwire associated with intermaxillary Class III elastics 16 hours a day.

Importantly, maxillary canines and second molars eruption was delayed, which increased total treatment time (41 months). Left mandibular second molar was mesially and buccolingually tipped while the patient and his parents were anxious for treatment conclusion. Thus, the appliance was completely removed with only the TMA rectangular archwire remaining on teeth #36 and 37 to correct #37 positioning. This wire segment was removed after 4 months when the patient brought his final exams to the last appointment. Additionally, the patient was referred to extraction of tooth #48 which was the only mesially tipped third molar he had.

A removable wraparound retainer was continuously used in the maxillary arch for 6 months during the day and at night for 2 years during the retention phase. In the mandibular arch, however, a 0.8-mm stainless steel wire intercanine bar was installed and used for life.

## RESULTS

**Figure 8:**
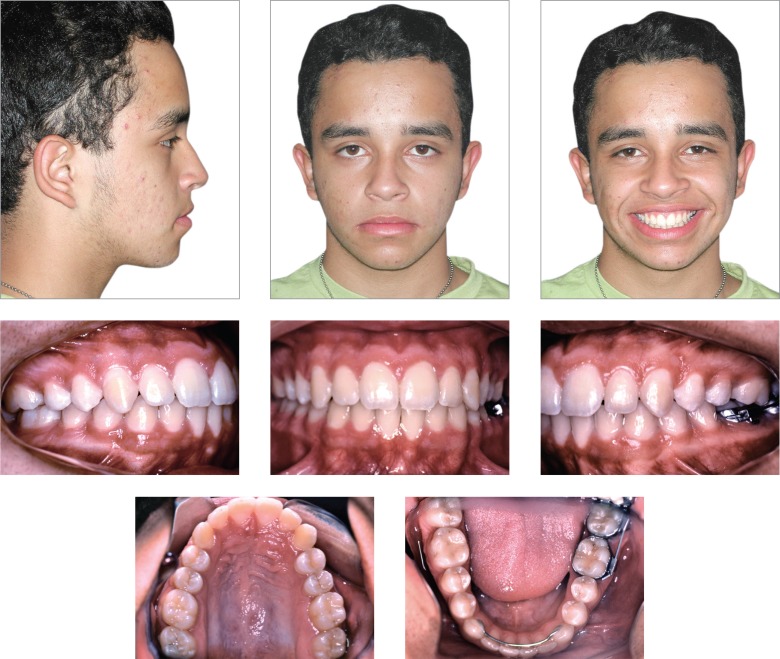
Final facial and intraoral photographs.

**Figure 9:**
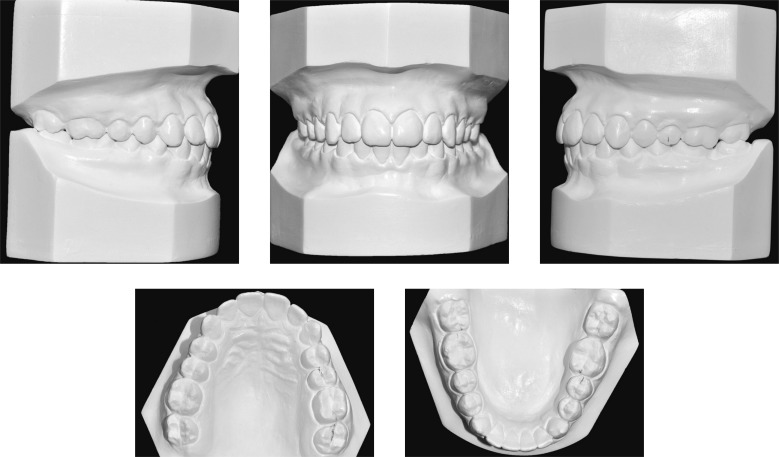
Final casts

**Figure 10:**
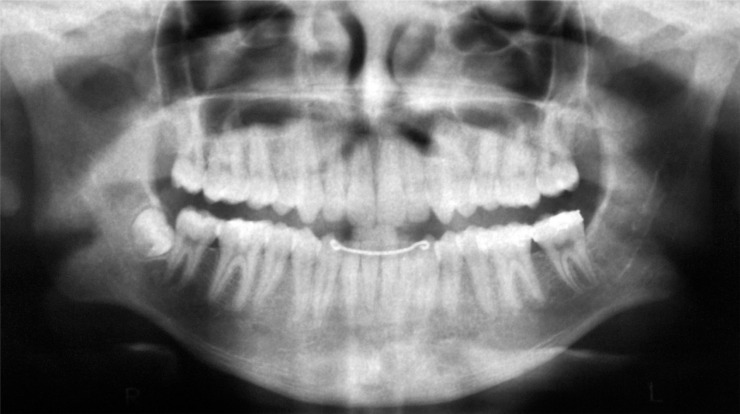
Final panoramic radiograph.

**Figure 11:**
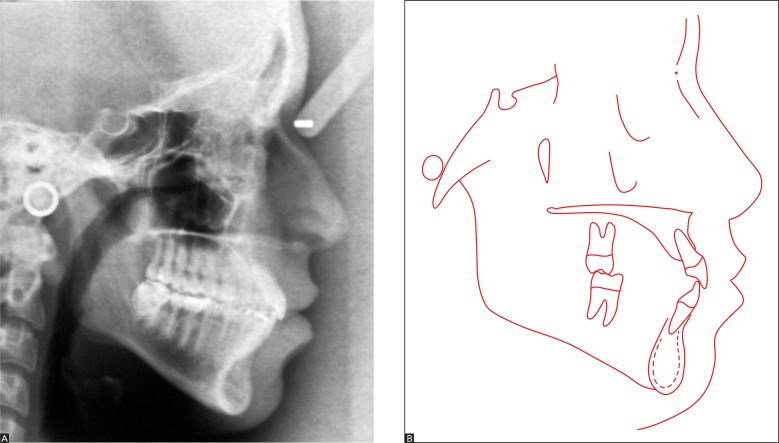
Final lateral cephalogram (A) and cephalometric tracing (B).

**Figure 12:**
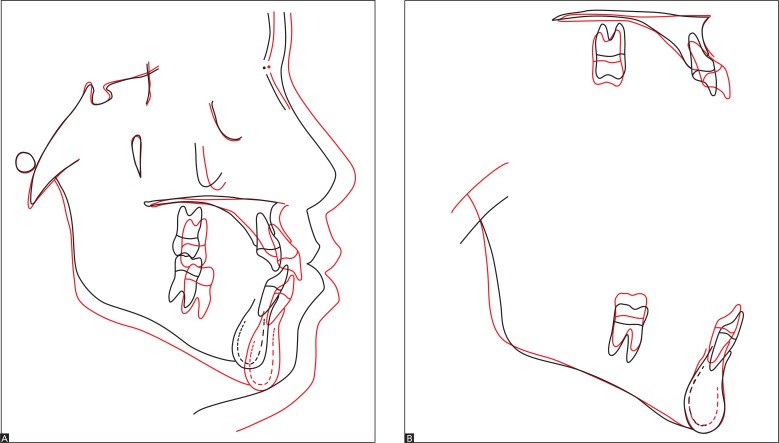
Initial (black) and final (red) cephalometric tracings total (A) and partial (B) superimposition.

Patient's final exams (Figs 8 to 12)revealed harmonious lip repositioning as well as improvements in facial profile which became slightly convex. Maxillomandibular relationship was restored to normality and ANB angle increased from 0.5^o^ to 2^o^. Such improvements were due to maxillary advancement (SNA increased in 2^o)^.Class III relationship was corrected by maxillary incisors buccal tipping (1-NA increased in 11^o^ and 3 mm).Mandibular incisors practically remained in their original position. Treatment was completed without further shifts between centric relation and maximum intercuspation. Occlusion guidance was restored (protrusion and laterality).

Cephalometric tracings superimposition (Fig 12A) revealed facial growth down and forward. Particularly due to protraction, the maxilla advanced towards the nasion point, thereby resulting in a balanced maxillomandibular relationship. Partial maxillary superimposition (Fig 12B)revealed buccal incisors tipping and little molar extrusion as a result of alveolar protraction and development expected for that period. Partial mandibular superimposition (Fig 12B) revealed mandibular growth as well as compensatory alveolar growth in molar and incisors regions.

In view of the above, it is reasonable to assert that treatment results were in accordance with treatment initial objectives. The patient was highly satisfied with his final facial and dental esthetics and remained aware of the need for a long-term follow-up to monitor mandibular growth and occlusal relationship.

**Figure 13:**
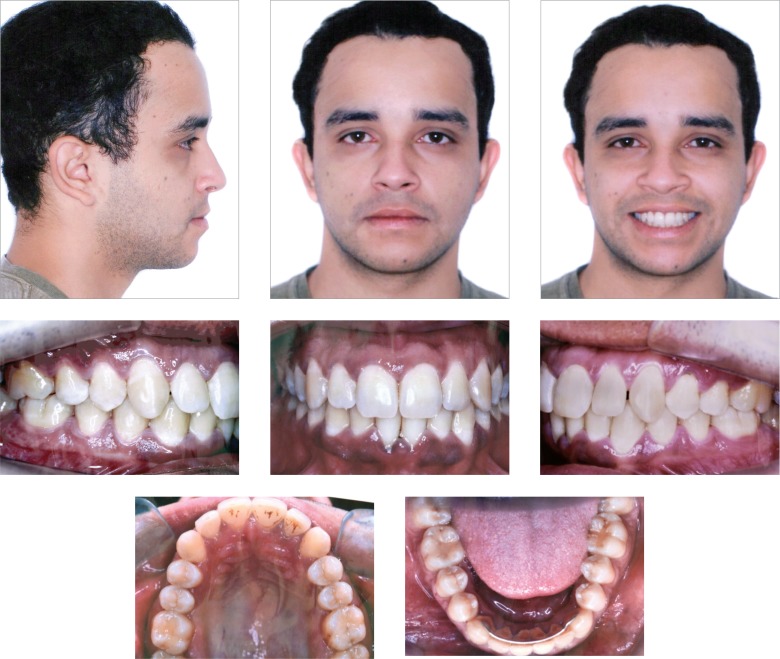
Control facial intraoral photographs 10 years after treatment completion.

**Figure 14:**
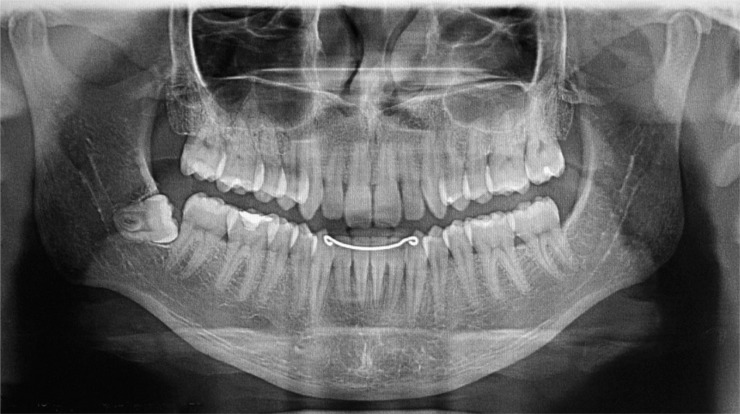
Control panoramic radiograph 10 years after treatment completion

**Figure 15:**
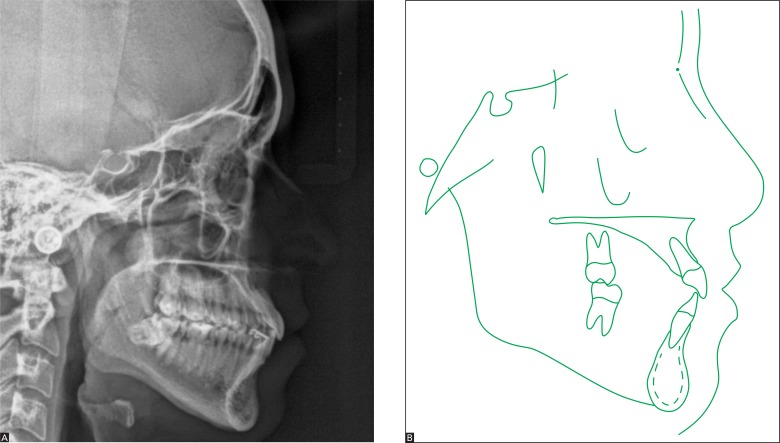
Control lateral cephalogram (A) and cephalometric tracing (B) 10 years after treatment completion.

**Figure 16:**
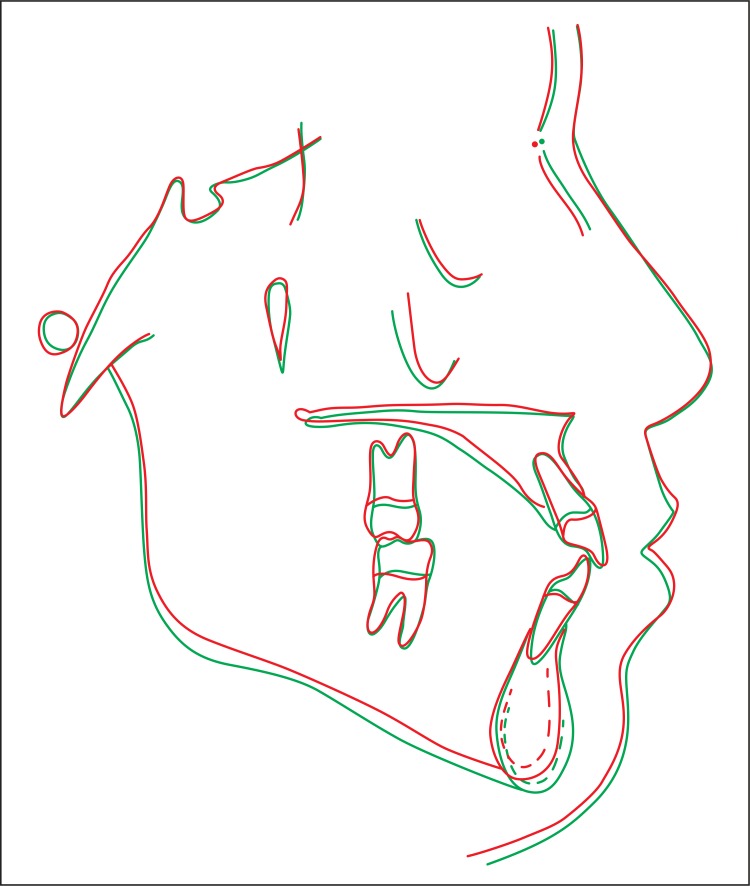
Final (red) and control cephalometric tracings superimposition 10 years after treatment (green).

**Figure 17:**
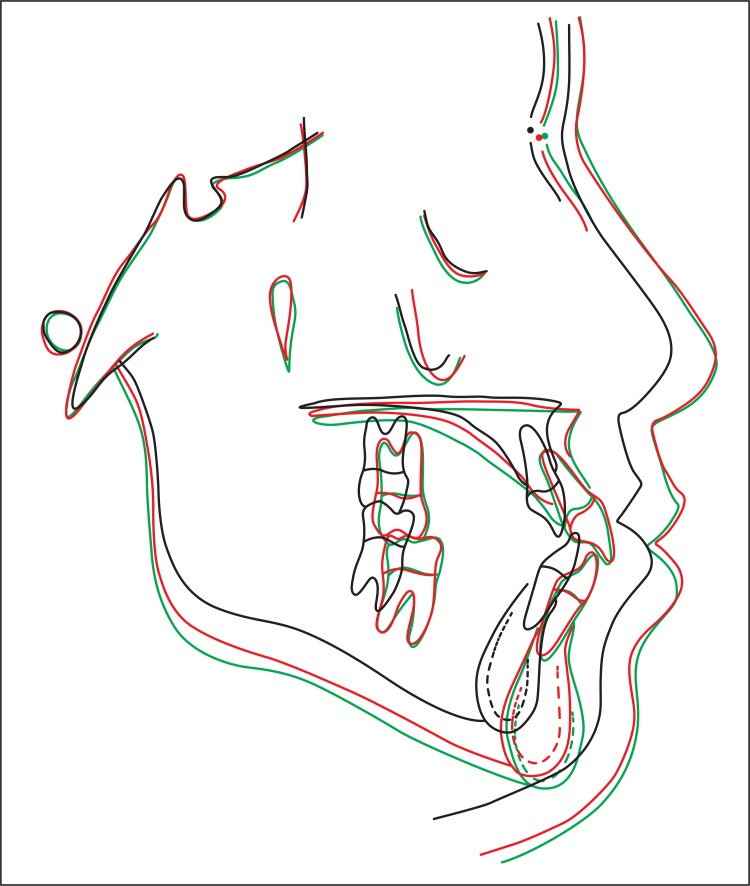
Initial (black), final (red) and control cephalometric tracings superimposition 10 years after treatment (green).

Total treatment time was long due to early treatment approach, delayed maxillary canines and second molars eruption as well as patient's absence at some appointments. New examinations were requested ten years after orthodontic treatment completion (Figs 13 to 17). They revealed excellent occlusal stability and facial balance. Both panoramic radiograph (Fig 14) and lateral cephalogram (Fig 15A) revealed all aspects were within standards of normality. The patient was advised in terms of periodontal care and the need for extracting tooth #48. Control and cephalometric tracings superimposition (Fig 16) revealed little residual mandibular growth which did not compromise facial and dental features achieved at treatment completion.

## FINAL CONSIDERATIONS

Class III skeletal malocclusion relies on early treatment approach to achieve treatment success without the need for surgery during adulthood.^2^ Early treatment with protraction of the maxilla has proved effective particularly in cases involving maxillary retrusion, as diagnosis of Class III, and meso or brachyfacial facial pattern. It results in adult stability in 75% of cases.^2^^,^ 3 Treatment is considered as early when performed before permanent dentition onset, in which case better results with small chances of relapse occur in patients younger than 10 years of age.^4^^,^ 7 In general, rapid maxillary expansion is associated with protraction of the maxilla,^8^^,^ 11 although the latter might be dispensable.^12^

The case reported herein revealed excellent stability due to patient's good growth pattern associated with appropriate intervention. Despite excellent stability, patients similar to the one reported in the present study must be informed about the potential need for compensatory retreatment and surgery.
